# Differential role of beta-arrestin ubiquitination in agonist-promoted down-regulation of M_1 _*vs *M_2 _muscarinic acetylcholine receptors

**DOI:** 10.1186/1750-2187-3-20

**Published:** 2008-12-03

**Authors:** Valerie A Mosser, Kymry T Jones, Katie M Hoffman, Nael A McCarty, Darrell A Jackson

**Affiliations:** 1 Department of Biomedical and Pharmaceutical Sciences, College of Health Professions and Biomedical Science, Skaggs Building Room 376, The University of Montana, Missoula, MT 59812, USA; 2 School of Biology, Georgia Institute of Technology, Atlanta, GA, 30332, USA

## Abstract

**Background:**

Sustained agonist-promoted ubiquitination of β-arrestin has been correlated with increased stability of the GPCR – β-arrestin complex. Moreover, abrogation of β-arrestin ubiquitination has been reported to inhibit receptor internalization with minimal effects on receptor degradation.

**Results:**

Herein we report that agonist activation of M_1 _mAChRs produces a sustained β-arrestin ubiquitination but no stable co-localization with β-arrestin. In contrast, sustained ubiquitination of β-arrestin by activation of M_2 _mAChRs does result in stable co-localization between the M_2 _mAChR and β-arrestin. Internalization of receptors was unaffected by proteasome inhibitors, but down-regulation was significantly reduced, suggesting a role for the ubiquitination machinery in promoting down-regulation of the receptors. Given the ubiquitination status of β-arrestin following agonist treatment, we sought to determine the effects of β-arrestin ubiquitination on M_1 _and M_2 _mAChR down-regulation. A constitutively ubiquitinated β-arrestin 2 chimera in which ubiquitin is fused to the C-terminus of β-arrestin 2 (YFP-β-arrestin 2-Ub) significantly increased agonist-promoted down-regulation of both M_1 _and M_2 _mAChRs, with the effect substantially higher on the M_2 _mAChR. Based on this observation, we were interested in examining the effects of disruption of potential ubiquitination sites in the β-arrestin sequence on receptor down-regulation. Agonist-promoted internalization of the M_2 _mAChR was not affected by expression of β-arrestin lysine mutants lacking putative ubiquitination sites, β-arrestin 2^K18R, K107R, K108R, K207R, K296R^, while down-regulation and stable co-localiztion of the receptor with this β-arrestin lysine mutant were significantly reduced. Interestingly, expression of β-arrestin 2^K18R, K107R, K108R, K207R, K296R ^increased the agonist-promoted down-regulation of the M_1 _mAChR but did not result in a stable co-localiztion of the receptor with this β-arrestin lysine mutant.

**Conclusion:**

These findings indicate that ubiquitination of β-arrestin has a distinct role in the differential trafficking and degradation of M_1 _and M_2 _mAChRs.

## Background

There are five subtypes of muscarinic acetylcholine receptors (M_1 _– M_5 _mAChR) with distinct yet overlapping tissue distributions. Muscarinic receptors regulate a variety of physiological responses ranging from cardiac homeostasis to cholinergic signaling in the brain [1]. A common feature of mAChRs, and in fact all GPCRs, is that both their expression and activation are tightly regulated. Agonist-promoted trafficking of mAChRs, and most other GPCRs can be broken down into five distinct phases: agonist-binding promotes G protein dissociation (I) from the receptor which allows phosphorylation of specific serine and threonine residues (II) on internal loops of the receptor by G protein receptor kinases (GRKs). This phosphorylation allows the binding of β-arrestins (III) which promotes homologous desensitization and subsequent internalization of the receptor into clathrin coated pits. Following internalization, the receptor can either be dephosphorylated and recycled (IV) to the cell surface or targeted for degradation (V) in proteasomes or lysosomes [1].

β-arrestins have emerged as a central control point in the trafficking of nearly all GPCRs [2]. In addition to mediating desensitization of GPCRs, β-arrestin also participates in clathrin-dependent endocytosis of activated receptors by directly interacting with clathrin and the clathrin-associated adaptor AP-2 [3,4]. Once internalized, β-arrestins are involved in regulation of post-endocytotic trafficking and recently have been shown to function as scaffolding proteins interacting with cellular trafficking machinery [2,5].

Initial reports examining the role of β-arrestin, clathrin and dynamin indicated that agonist-promoted internalization of the M_2 _mAChR proceeded via both arrestin-dependent and -independent pathways [6,7]. Later work, however, indicated the essential role of β-arrestin in M_2 _mAChR internalization, and suggested that all M_2 _mAChR internalization, like other mAChR subtypes, is dynamin-dependent [8,9].

Two classes of GPCRs have been identified with respect to kinetics of receptor recycling and interaction with β-arrestins. Class A receptors, such as the β_2 _adrenergic (β_2_AR), dopamine D1A and endothelin 1A receptors recycle rapidly and dissociate from β-arrestin prior to receptor internalization. Class B receptors, such as the vasopressin 2 (V2R), angiotensin 1a and neurotensin 1 receptors, recycle slowly and internalize in a stable association with β-arrestin [10,11]. Recently, it has become clear that the classification of receptors as A or B directly correlates with patterns of β-arrestin ubiquitination and deubiquitination [12]. Stimulation of Class A β_2_ARs leads to transient ubiquitination of β-arrestin with deubiquitination occurring shortly (minutes) after internalization [12]. In contrast, stimulation of Class B V2Rs leads to a stable ubiquitination of β-arrestin [12].

Ubiquitination of proteins is a signal for degradation that leads to delivery to and degradation of proteins in the 26S proteasome [13]. There are a number of examples where ubiquitination has been shown to be involved in the regulation of GPCRs, including the opiod receptors [14], yeast pheromone receptor [15], human immunodeficiency virus co-receptor CXCR4 [16], and β_2 _ARs [17].

Class A receptors, which do not internalize with β-arrestin, display a pattern of transient β-arrestin ubiquitination. Class B receptors, on the other hand, do internalize with β-arrestin, and display a sustained β-arrestin ubiquitination pattern [12]. Shenoy *et al. *[17] showed that agonist stimulation led to the ubiquitination of both β-arrestin and the β_2 _AR. Their data suggested that it was the ubiquitination of β-arrestin that was involved in the initial receptor internalization step whereas ubiquitination of the receptor itself was required for subsequent receptor degradation.

In the present study, we wanted to address several questions regarding the role of β-arrestin ubiquitination in the agonist-promoted down-regulation of mAChRs. First we wanted to determine if there was a role for β-arrestin ubiquitination in receptor down-regulation. Collectively, our data support a role for β-arrestin in differential targeting of mAChRs towards down-regulation. In addition, the data indicate that this differential targeting may be controlled by preferential interaction of mAChR subtypes with the different β-arrestin subtypes which in turn may be mediated by specific patterns of β-arrestin ubiquitination.

## Results

For a number of years there has been some controversy as to the role of β-arrestin in M_2 _mAChR specific internalization and down-regulation. Our recent publication [9] established the essential role of β-arrestin in the internalization of the M_2 _mAChR. The current study is a follow up which not only addresses the role of β-arrestin in agonist-promoted mAChR down-regulation, but also indicates a differential role for β-arrestin in M_1 _versus M_2 _mAChR down-regulation. As in the previous study, we performed our down-regulation experiments in mouse embryonic fibroblasts (MEFs) derived from β-arrestin 1/2 double knockout mice (MEF KO1/2) [18]. Experiments performed using this cell line offer a significant advantage over previous studies of the role of β-arrestin in GPCR internalization and down-regulation that used dominant-negative or knockdown strategies because they avoid any possible complications that could arise from the presence of low levels of endogenous arrestin proteins. These cells have been characterized to confirm the absence of mAChR expression using both PCR and radioligand binding assays [9].

**Figure 1:**
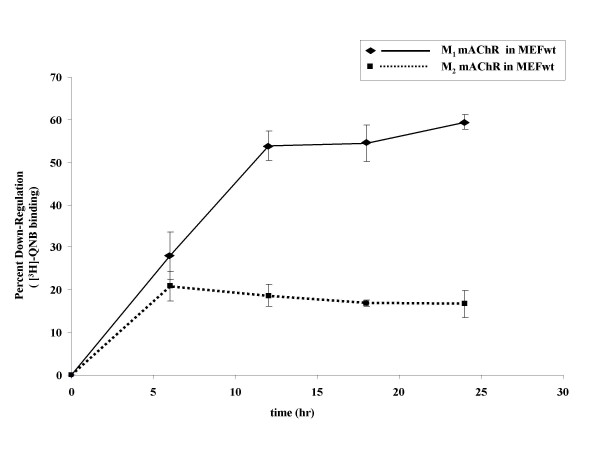
**Time-course of agonist-promoted down-regulation of mAChRs in MEF**. MEF wt cells were transfected with eGFP-M_1 _(◆) or HA-M_2 _(■) mAChR. 24 hr following transfection, cells were treated with 1 mM carbachol for the indicated time. Down-regulation was determined using [^3^H]-QNB binding (fmol/mg protein) in crude membranes as described in methods. The M_1 _mAChR displays nearly threefold the down-regulation of the M_2 _subtype over the same time course. Data are expressed as percent down-regulation compared to t = 0 control and are presented as mean ± standard error of the mean for three independent experiments with duplicate data points. Total mAChR expressed (fmol/mg) at t = 0 was 4000 for both M_1 _and M_2 _mAChRs.

To determine whether or not MEF cells could be used in the current study, we performed agonist-promoted down-regulation time courses for both the M_1 _and M_2 _mAChR subtypes. Total receptor numbers were measured using the non-selective membrane permeable muscarinic antagonist quinuclidinyl benzilate (QNB), which binds to both surface and intracellular pools of mAChR. Wild-type MEF cells that were transfected with either eGFP-M_1 _mAChR or HA-M_2 _mAChR resulted in similar expression levels (~4000 fmol/mg). After 24 hr, cells were treated with 1 mM carbachol for the indicated time. Maximal down-regulation of the M_2 _mAChR occurred after 6 hr of stimulation (Figure 1). In contrast, maximal down-regulation of the M_1 _subtype did not occur until after 12 hr of carbachol stimulation. In addition, M_2 _mAChRs were maximally decreased by only 22% while the M_1 _subtype was decreased by 55%. These results demonstrate that exogenously expressed M_1 _and M_2 _mAChRs undergo agonist-promoted down-regulation in MEFwt cells. The different time course and extent of down-regulation suggest the possibility that down-regulation of the two subtypes may involve distinct pathways. Despite the fact that M_2 _mAChRs were maximally down-regulated by 6 hr of stimulation, subsequent single time point experiments used the 12 hr time point so that the M_1 _and M_2 _subtype experiments would be compared at a standardized time point.

**Figure 2:**
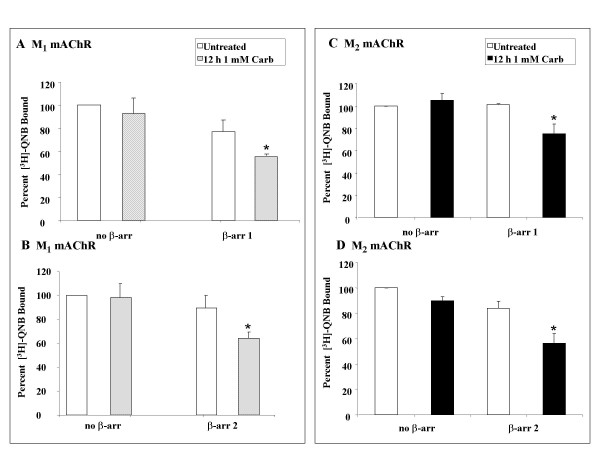
**Rescue of mAChR down-regulation in MEF KO1/2 with β-arrestin 1 or 2**. MEF KO1/2 cells were transfected with eGFP-M_1 _mAChR (**A-B**) or HA-M_2 _mAChR (**C-D**) and either FLAG-β-arrestin 1 (**top**) or 2 (**bottom**). 24 hr following transfection, cells were treated with 1 mM carbachol for 12 hr. Down-regulation was determined using [^3^H]-QNB binding (fmol/mg protein) in crude membranes as described in methods. No down-regulation occurs in the absence of β-arrestin and either isoform (β-arrestin 1 or 2) rescues both constitutive and agonist-promoted down-regulation. Data are expressed as percent of [^3^H]-QNB bound (fmol/mg total protein) compared to untreated, no β-arrestin control and are presented as mean ± standard error of the mean from three independent experiments with duplicate data points. Statistical analysis was performed using a paired t-test. *: p ≤ 0.05 versus the untreated, no β-arrestin control. Total M_1 _mAChR expressed (fmol/mg) at t = 0 was 400 – 900 (M_1_) and 1000 – 2000 (M_2_) for both the β-arrestin 1 and β-arrestin 2 rescue experiments.

Several recent studies have reported that mAChR down-regulation occurs independently of agonist-promoted internalization [19-21]. Recently, we demonstrated that agonist-promoted internalization of M_2 _mAChRs in MEF cells was β-arrestin dependent [9]. To examine whether or not agonist-promoted down-regulation of M_1 _and M_2 _mAChRs is β-arrestin dependent, we performed down-regulation studies in MEF β-arrestin double knockout cells transiently expressing either M_1 _or M_2 _mAChRs. MEF KO1/2 cells were transfected with eGFP-M_1 _mAChR or HA-M_2 _mAChR and either FLAG-β-arrestin 1 or 2. After 24 hr, cells were treated with 1 mM carbachol for 12 hr. In the absence of exogenous β-arrestin, there was no down-regulation in response to agonist stimulation in the double knockout cell line (Figure 2A–D). Expression of either β-arrestin 1 or β-arrestin 2, however, rescued down-regulation of both receptor subtypes in response to agonist (Figure 2A – D). These results demonstrate that agonist-promoted down-regulation of M_1 _and M_2 _mAChRs is β-arrestin dependent.

**Figure 3:**
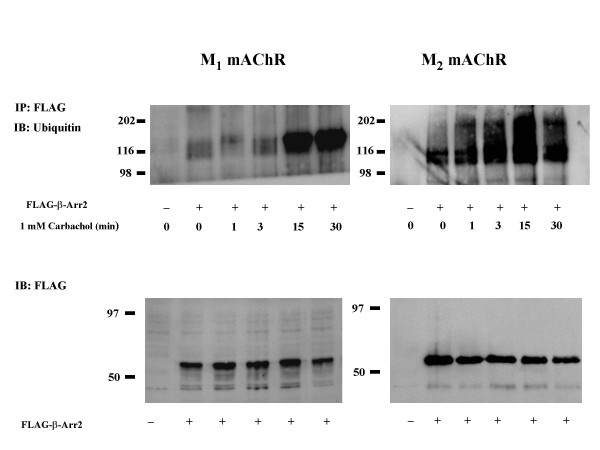
**Agonist treatment promotes ubiquitination of β-arrestin 2 in MEF KO1/2 cells expressing the mAChRs and β-arrestin 2**. MEF KO1/2 cells transfected with FLAG-β-arrestin 2 and either eGFP-M_1 _or HA-M_2 _mAChR. 24 hr following transfection, cells were treated with 1 mM carbachol for the indicated times. Top panel: cells were lysed and immunoprecipitated (IP) with anti-FLAG MAb and blotted (IB) with anti-ubiquitin-MAb. There was an increase in ubiquitination of β-arrestin 2 with increasing exposure to 1 mM carbachol. Each blot is representative of two independent experiments. **Bottom panel**: lysates blotted with anti-FLAG MAb to demonstrate β-arrestin 2 expression levels.

Recent studies have demonstrated that agonist stimulation of GPCRs results in ubiquitination of β-arrestin 2 [12]. This group demonstrated receptor specific agonist-promoted ubiquitination of β-arrestin 2 that was either transient (peaked at 1 min of agonist stimulation) or stable (still present after 15 min of agonist stimulation) [12]. We performed experiments in order to determine if agonist stimulation of M_1 _or M_2 _mAChRs promoted the ubiquitination of β-arrestin and if so, examine whether the observed ubiquitination was transient or stable. MEF KO1/2 cells were transfected with FLAG-β-arrestin 2 and eGFP-M_1 _or HA-M_2 _mAChR and treated with carbachol for 0, 1, 3, 15 and 30 min. Stimulation of M_1 _and M_2 _mAChRs significantly increased ubiquitination of β-arrestin 2 (Figure 3). This increase is clearly visible at both the 15 and 30 min time point for both mAChR subtypes. These results demonstrate that stimulation of M_1 _or M_2 _mAChRs promotes a stable ubiquitination of β-arrestin 2.

**Figure 4:**
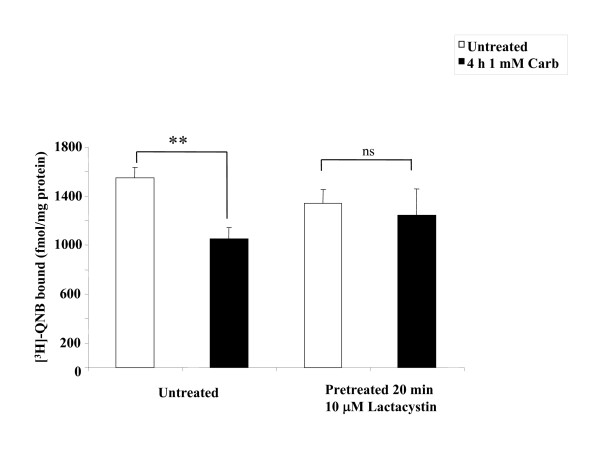
**The proteosomal inhibitor lactacystin inhibits the β-arrestin 2 mediated agonist-promoted down-regulation of the M_2 _mAChR in MEFwt**. MEFwt cells were transfected with HA-M_2 _mAChR and, after 24 hr cells were incubated for 20 min with or without 10 μM lactacystin then treated with 1 mM carbachol for 4 hr. Lactacystin inhibits the β-arrestin 2 mediated down-regulation of the M_2 _mAChR receptor in response to agonist. Down-regulation was determined using [^3^H]-QNB binding in crude membranes as described in methods. Data are expressed as [^3^H]-QNB bound (fmol/mg total protein) and are presented as mean ± standard deviation from three independent experiments with duplicate data points. Statistical analysis was performed using a paired t-test, ** indicates p ≤ 0.001 (compared to paired untreated control), ns indicates not significant.

Having established that β-arrestin is required for mAChR down-regulation and that muscarinic stimulation leads to the ubiquitination of β-arrestin, we wanted to examine if there was a direct role for β-arrestin ubiquitination in receptor down-regulation. Since ubiquitination is known to serve as a signal for protein degradation [13] we were interested in determining whether disruption of the ubiquitin/proteasome pathway would affect the agonist-promoted down-regulation of the mAChRs. To confirm that β-arrestin 2 promotes down-regulation via a ubiquitin dependent pathway, we examined agonist-promoted down-regulation of the M_2 _mAChR in the presence of the proteosomal inhibitor lactacystin (impairs ubiquitin recycling). MEFwt cells were transfected with HA-M_2 _mAChR and after 24 hr cells were incubated for 20 min with or without 10 μM lactacystin prior to treatment with 1 mM carbachol for 4 hr. Lactacystin was able to completely block agonist-promoted down-regulation (Figure 4) suggesting that the ubiquitination machinery has a role in mediating agonist-promoted down-regulation of mAChRs.

**Figure 5:**
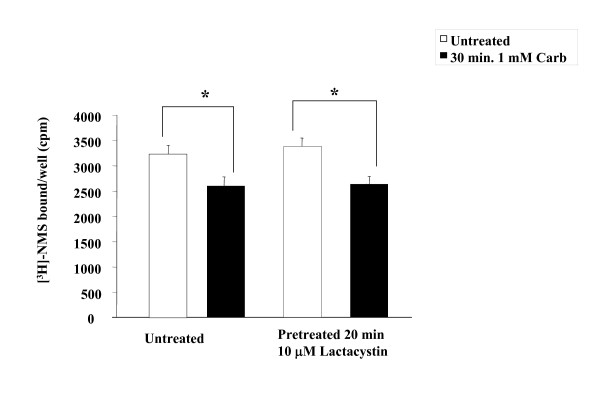
**The proteosomal inhibitor lactacystin does not affect agonist-promoted down-regulation internalization of the M_2 _mAChR in MEFwt**. MEFwt cells were transfected with HA-M_2 _mAChR and, after 24 hr cells were incubated for 20 min with or without 10 μM lactacystin then treated with 1 mM carbachol for 30 min. Lactacystin has no effect on the internalization of the M_2 _mAChR receptor in response to agonist. Internalization was determined using [^3^H]-NMS binding in whole cells as described in methods. Data are [^3^H]-NMS bound per well (plated at 1 × 10^5 ^cells) and are presented as mean ± standard deviation from three independent experiments with duplicate data points. Statistical analysis was performed using a paired t-test, * indicates p ≤ 0.05; (compared to paired untreated control), ns indicates not significant.

To determine whether or not the effects of lactacystin were occurring at the level of receptor internalization, we examined the effect of lactacystin on the agonist-promoted internalization of mAChRs using the membrane impermeable muscarinic antagonist *N*-methylscopolamine (NMS). MEFwt cells were transfected with HA-M_2 _mAChR, and after 24 hr cells were incubated for 20 min in the absence or presence of 10 μM lactacystin prior to treatment with 1 mM carbachol for 30 min. Pretreatment with inhibitor had no effect on agonist-promoted internalization (Figure 5). These results indicate that agonist-promoted down-regulation of mAChRs involves receptor β-arrestin ubiquitination.

**Figure 6:**
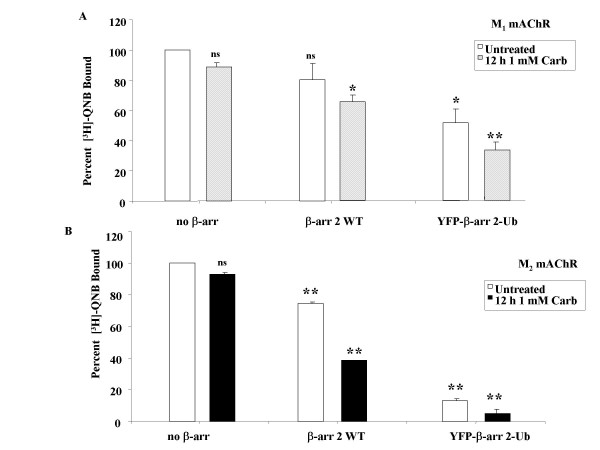
**Expression of a chimeric ubiquitinated form of β-arrestin 2 enhances the agonist-promoted down-regulation of M_1 _and M_2 _mAChRs in MEF KO1/2**. MEF KO1/2 cells were transfected with eGFP-M_1 _(**A**) or HA-M_2 _(**B**) mAChRs and either no β-arrestin, FLAG-β-arrestin 2 or YFP-β-arrestin 2-Ub. 24 hr following transfection, cells were treated with 1 mM carbachol for 12 hr. Down-regulation was determined using [^3^H]-QNB binding (fmol/mg protein) in crude membranes as described in methods. Expression of the constitutively ubiquitinated form of β-arrestin (β-arrestin 2-Ub) had a greater effect on down-regulation of the M_2 _mAChR compared to the M_1 _subtype. Data are expressed as percent of [^3^H]-QNB bound compared to the untreated control with no β-arrestin and are presented as mean ± standard deviation from two independent experiments with duplicate data points. Statistical analysis was performed using a repeated measures ANOVA with Bonferroni post test; * indicates p ≤ 0.05 and ** indicates p ≤ 0.001 (compared to untreated, no β-arrestin control), ns indicates not significant. Total mAChR expressed (fmol/mg) in the absence of β-arrestin was 1000 – 2000 for both receptor subtypes.

**Figure 7:**
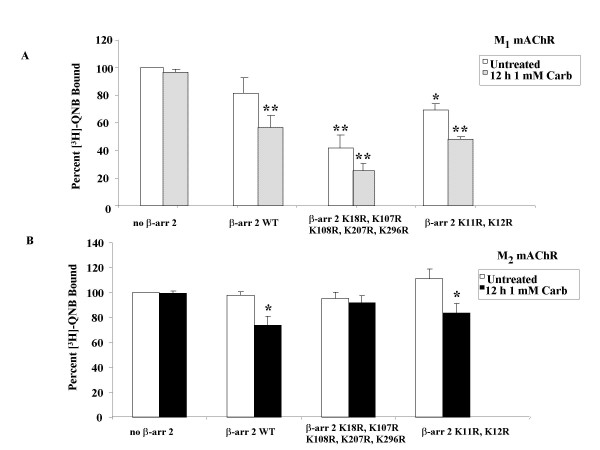
**β-arrestin 2^K18R, K107R, K108R, K207R, K296R^differentially affects down-regulation of M_1 _and M_2 _mAChRs in MEF KO1/2**. MEF KO1/2 cells were transfected with eGFP-M_1 _(**A**) mAChR or HA-M_2 _(**B**) mAChR and either empty vector (control), FLAG-β-arrestin 2 (WT), FLAG-β-arrestin 2^K18R, K107R, K108R, K207R, K296R ^or FLAG-β-arrestin 2^K11R, K12R^. 24 hr after transfection, cells were treated with 1 mM carbachol for 12 hr. Down-regulation was determined in crude membranes (fmol/mg protein) as described in methods. All β-arrestin 2 constructs were able to mediate agonist-promoted down-regulation of mAChR with the exception of the β-arrestin 2^K18R, K107R, K108R, K207R, K296R ^mutant when co-expressed with the M_2 _mAChR. Data are expressed as percent of [^3^H]-QNB bound (compared to untreated, no β-arrestin control) and presented as mean ± standard deviation from three independent experiments with duplicate or quadruplicate data points. Statistical analysis was performed using a repeated measures ANOVA with Bonferroni post test; * indicates p ≤ 0.05 ** indicates p ≤ 0.001 (compared to untreated, no β-arrestin control), ns indicates not significant. Total M_1 _(300–500 fmol/mg) and M_2 _(1500–2500 fmol/mg) mAChR expressed in the absence of β-arrestin constructs was similar.

To examine the consequences of β-arrestin ubiquitination on receptor down-regulation, we expressed a yellow fluorescent protein-tagged β-arrestin 2-ubiquitin chimera that cannot be deubiquitinated by cellular deubiquitinases (YFP-β-arrestin 2-Ub) [12]. MEF KO1/2 cells were transfected with eGFP-M_1 _or HA-M_2 _mAChR and either FLAG-β-arrestin 2 or YFP-β-arrestin 2-Ub. After 24 hr, cells were treated with 1 mM carbachol for 12 hr. There was a 40% agonist-promoted down-regulation of the M_1 _mAChR by β-arrestin 2 alone which increased to 70% in the presence of β-arrestin 2-Ub (Figure 6). These values were 62 and 95%, respectively, for the M_2 _subtype (Figure7). β-arrestin 2-Ub also increased the constitutive receptor down-regulation, and again the effect was much larger for the M_2 _(90%) *vs *M_1 _(50%) subtype (Figure 6A and 6B). It is clear from these data that ubiquitination enhanced the ability of β-arrestin 2 to mediate both constitutive and agonist-promoted down-regulation of both the M_1 _and M_2 _mAChRs.

Having demonstrated a role for ubiquitin in agonist-promoted mAChR degradation, we were interested in the effects of disrupting β-arrestin 2 ubiquitination on receptor down-regulation. Several lysine residues on β-arrestin are known to be sites of ubiquitination [22]. To further confirm the essential role of ubiquitination in agonist-promoted down-regulation, we examined the ability of specific β-arrestin 2 lysine mutants to mediate agonist-promoted down-regulation of the M_1 _and M_2 _mAChR. MEF KO1/2 cells were transfected with eGFP-M_1 _or HA-M_2 _mAChR and either empty vector (control), FLAG-β-arrestin 2 (wild-type), FLAG-β-arrestin 2^K18R, K107R, K108R, K207R, K296R ^or FLAG-β-arrestin 2^K11R, K12R^. After 24 hr, cells were treated with 1 mM carbachol for 12 hr. As shown previously, there was no down-regulation in the control cells in the absence of β-arrestin 2 (Figure 7). All three β-arrestin 2 constructs were able to rescue agonist-promoted down-regulation of the M_1 _subtype. We also noted a large constitutive effect on down-regulation with both mutant β-arrestins. In contrast, for the M_2 _subtype only wild-type β-arrestin 2 (24%) and β-arrestin 2^K11R, K12R ^(27%) were able to rescue agonist-promoted down-regulation (Figure 7). β-arrestin 2^K18R, K107R, K108R, K207R, K296R^, had lost the ability to rescue agonist-promoted down-regulation of the M_2 _mAChR (Figure 7).

**Figure 8:**
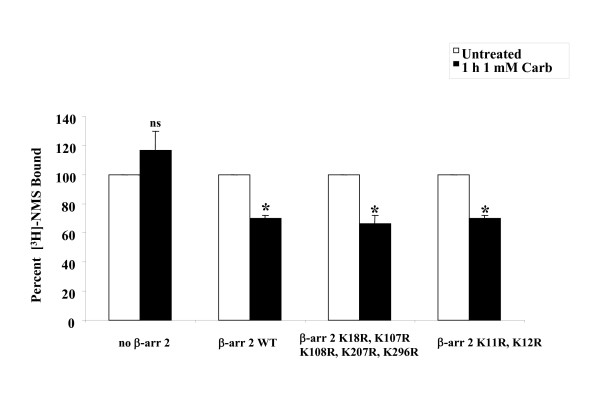
**Agonist-promoted internalization of M_2 _mAChR is unaffected by β-arrestin 2 lysine mutants in MEF KO1/2**. MEF KO1/2 cells were transfected with HA-M_2 _mAChR and either empty vector (control), FLAG-β-arrestin 2 (WT), FLAG-β-arrestin 2^K18R, K107R, K108R, K207R, K296R ^or FLAG-β-arrestin 2^K11R, K12R^. 24 hr after transfection, cells were treated with 1 mM carbachol for 1 hr. Internalization was determined in whole cells (receptors/cell) as described in methods. All constructs were able to mediate agonist-promoted internalization. Data are expressed as percent of [^3^H]-NMS bound compared to untreated control and presented as mean ± standard deviation from three independent experiments with duplicate data points. Statistical analysis was performed using a repeated measures ANOVA with Bonferroni post test; * indicates p ≤ 0.05 (compared to untreated, no β-arrestin control), ns indicates not significant. Total receptor expressed in the presence of all four β-arrestin constructs was between 5 and 6 × 10^5 ^receptor/cell.

Since it was possible that the effect of β-arrestin 2^K18R, K107R, K108R, K207R, K296R ^on M_2 _mAChR down-regulation seen in the previous experiment was actually occurring at the receptor internalization step, we performed receptor internalization experiments with [^3^H]-NMS. Transfections were identical to the previous experiment. After 24 hr, cells were treated with 1 mM carbachol for 1 hr. All β-arrestin 2 constructs (wild-type or lysine mutants) were able to rescue agonist-promoted internalization of M_2 _mAChRs in MEF KO1/2 (Figure 8).

**Figure 9:**
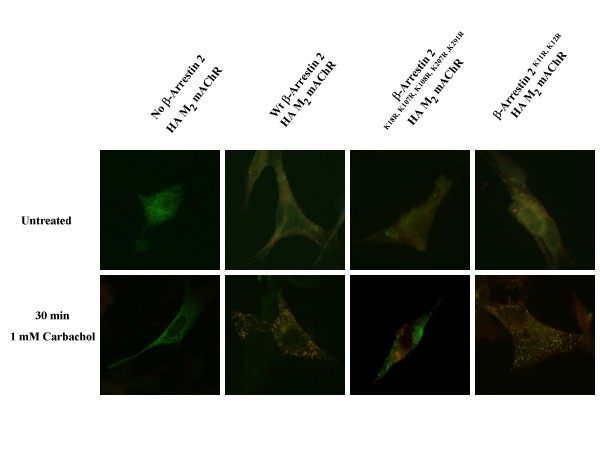
**β-arrestin 2^K18R, K107R, K108R, K207R, K296R^demonstrates impaired agonist-promoted co-localiztion with M_2 _mAChR in MEF KO1/2**. MEF KO1/2 cells were transfected with HA-M_2 _mAChRs (green) and either wild-type FLAG-β-arrestin 2, FLAG-β-arrestin 2^K18R, K107R, K108R, K207R, K296R ^or FLAG-β-arrestin 2^K11R, K12R ^(red). 24 hr after transfection, cells were treated with 1 mM carbachol for 30 min. Cells were fixed, probed, and imaged as described in methods. Wild-type and β-arrestin 2^K11R, K12R ^demonstrate co-localization with the M_2 _mAChR which is significantly diminished with β-arrestin 2^K18R, K107R, K108R, K207R, K296R^. Image shown is representative of two independent experiments.

**Figure 10:**
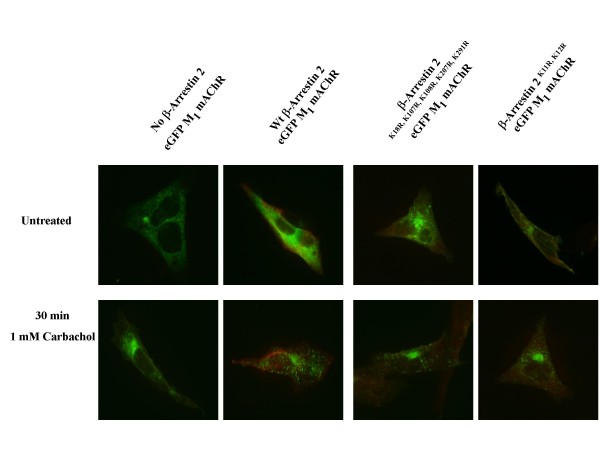
**β-arrestin 2^K18R, K107R, K108R, K207R, K296R^demonstrates no co-localization with M_1 _mAChR in MEF KO1/2**. MEF KO1/2 cells were transfected with eGFP-M_1 _(A) mAChRs (green) and either wild-type FLAG-β-arrestin 2, FLAG-β-arrestin 2^K18R, K107R, K108R, K207R, K296R ^or FLAG-β-arrestin 2^K11R, K12R ^(red). 24 hr after transfection, cells were treated with 1 mM carbachol for 30 min. Cells were fixed, probed, and imaged as described in methods. None of the β-arrestin constructs demonstrate co-localization with the M_1 _mAChR. Image shown is representative of two independent experiments.

Since β-arrestin 2^K18R, K107R, K108R, K207R, K296R ^not only appears to interfere with the agonist promoted down-regulation of the M_2 _mAChR but also increased the constitutive down-regulation of the M_1 _mAChR we examined the ability of this mutant to co-localize with the M_1_/M_2 _mAChR compared to wt and β-arrestin 2^K11R, K12R ^following agonist stimulation. MEF KO1/2 were plated on cover slips in 6-well plates. 24 hrs after transfection with eGFP-M_1 _mAChR or HA-M_2 _mAChR and FLAG tagged β-arrestin (wt or lysine mutants) cells were treated for 30 min or 12 hrs with 1 mM carbachol. Cells were fixed and processed for indirect immunofluorescence as described in methods. After 30 minutes of agonist exposure there is clear overlap of the M_2 _mAChR receptor and β-arrestin signal signified by the yellow puncta (Figure 9) for the wt and β-arrestin 2^K11, K12R^. This overlap is absent with β-arrestin 2^K18R, K107R, K108R, K207R, K296R ^which indicates a disrupted or impaired interaction between this β-arrestin mutant and the M_2 _mAChR. Surprisingly, despite our previous observation of the effects of β-arrestin 2^K18R, K107R, K108R, K207R, K296R ^on constitutive M_1 _down-regulation, no co-localization was observed between β-arrestin and the M_1 _mAChR (Figure 10).

**Figure 11:**
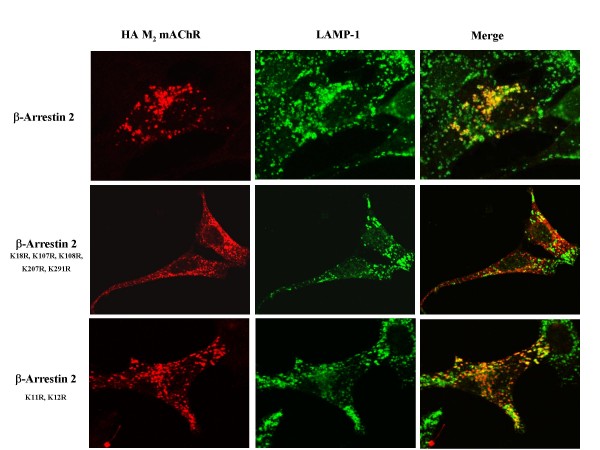
**β-arrestin 2^K18R, K107R, K108R, K207R, K296R^disrupts the co-localization of M_2 _mAChR and LAMP-1 in MEF KO1/2**. MEF KO1/2 cells were transfected with HA-M_2 _mAChR and either wild-type FLAG-β-arrestin 2, FLAG-β-arrestin 2^K18R, K107R, K108R, K207R, K296R ^or FLAG-β-arrestin 2^K11R, K12R^. 24 hr after transfection, cells were treated with 1 mM carbachol for 6 hr. Cells were fixed, probed, and imaged as described in methods. β-arrestin 2^K18R, K107R, K108R, K207R, K296R ^disrupts the co-localization of M_2 _mAChR and LAMP-1 compared to wild-type and β-arrestin 2^K11R, K12R^. Image shown is representative of three independent experiments.

Finally we used immunocytochemistry with the lysosomal marker LAMP-1 to examine the effects of the β-arrestin 2^K18R, K107R, K108R, K207R, K296R ^mutant on mAChR lysosomal targeting down-regulation. MEF KO1/2 were transfected with HA-M_2 _mAChR and either empty vector (control), FLAG-β-arrestin 2 (wild-type), FLAG-β-arrestin 2^K11R, K12R ^or FLAGβ-arrestin 2^K18R, K107R, K108R, K207R, K296R^. After 24 hr, cells were treated with 1 mM carbachol for 6 hr. There was significant co-localization of the receptor with LAMP-1 in the presence of wild-type β-arrestin 2 or β-arrestin 2^K11R, K12R ^(Figure 11). It is clear, however, that β-arrestin 2^K18R, K107R, K108R, K207R, K296R ^shows reduced co-localization of the M_2 _mAChR with LAMP (Figure 11).

Collectively, these data indicate that the agonist-promoted down-regulation of the M_1 _and M_2 _mAChRs involves differential sorting of the receptor by ubiquitinated β-arrestin.

## Discussion

Our recent work established the essential role of β-arrestin in the internalization of the M_2 _mAChR [9]. The present study extends the observations of previous work and demonstrates that the agonist-promoted down-regulation of M_1 _and M_2 _mAChRs is β-arrestin dependent, and that the ubiquitination pattern of β-arrestin has a critical role in the differential down-regulation for M_1 _*vs *M_2 _mAChRs.

It has been previously established in a variety of cell lines that a prolonged activation of M_1 _or M_2 _mAChRs induces receptor down-regulation. In agreement with these findings, long-term stimulation of M_1 _or M_2 _mAChRs induced receptor down-regulation with the M_1 _mAChR showing significantly more down-regulation than M_2 _subtype. This observation suggests that the two subtypes are differentially regulated by endogenous β-arrestins. No down-regulation of either mAChR subtype occurred in the absence of β-arrestin and either β-arrestin subtype was able to rescue receptor down-regulation.

The observations of Shenoy and co-workers suggest that the ubiquitination of β-arrestin is a critical control point in the trafficking of different classes of GPCRs [12,17,22]. The time course of ubiquitination of β-arrestin 2 in response to muscarinic stimulation in MEF KO1/2 cells transiently expressing the M_1 _or M_2 _mAChRs was slow in onset and stable over time. This observation suggests that both M_1 _and M_2 _mAChRs display a Class B β-arrestin ubiquitination pattern despite the fact that the M_1 _mAChR is known to recycle rapidly compared to M_2 _[23], and does not show a stable endocytotic co-localization with β-arrestin 2 [9] – both characteristics of Class A GPCRs. This observation suggests that there is some flexibility in the Class A *vs *B distinction. This same flexibility has been observed in the categorization of somatostatin receptor subtypes [24].

To confirm that agonist-promoted down-regulation of mAChRs was ubiquitin-dependent we performed internalization/down-regulation experiments in the presence of the proteosomal inhibitor lactacystin. Lactacystin is a specific inhibitor of the 26S proteasome that functions by covalently modifying the active site and inhibiting the enzymatic activity of the proteasome [13]. This inhibition of the enzyme would inhibit recycling and thus deplete the available ubiquitin. Lactacystin was able to completely block agonist-promoted down-regulation with no effect on agonist-promoted internalization. These results suggested that the availability of free ubiquitin has a role in agonist-promoted down-regulation of the mAChRs. This effect is most likely due to effects on ubiquitin-dependent lysosomal sorting as evidenced by a later experiment that demonstrated the co-localization of the agonist-internalized M_2 _mAChR with the lysosomal membrane protein LAMP-1.

Having established that β-arrestin is ubiquitinated via mAChR activation, we then examined whether the ubiquitination of β-arrestin enhances its ability to mediate agonist-promoted down-regulation. M_1 _mAChR levels were reduced and M_2 _levels were nearly completely ablated in the presence of β-arrestin 2-Ub compared to wild-type β-arrestin. The effect was seen on both constitutive and agonist promoted down-regulation.

In order to examine the role of β-arrestin ubiquitination in targeting mAChRs toward degradation, we used β-arrestin lysine mutants that have been previously shown to have a role in the endocytotic trafficking of other GPCRs. The constructs, generated by Shenoy and co-workers, contain mutations of potential ubiquitination sites near the amino terminus (β-arrestin 2^K11R, K12R^) and at five other sites in the protein (β-arrestin 2^K18R, K107R, K108R, K207R, K296R^) [22]. We were particularly interested in the possible differential effects of β-arrestin ubiquitination patterns on M_1 _*vs *M_2 _down-regulation given the fact that M_1 _and M_2 _mAChRs did not fit neatly into the classical Class A *vs *B distinction.

Both of the β-arrestin lysine mutants were able to mediate agonist-promoted down-regulation of the M_1 _mAChR to the same extent as wild-type β-arrestin. While our data clearly indicates an essential role for β-arrestin ubiquitination in M_1 _mAChR down-regulation, other lysine residues than the ones examined must be the targets for the necessary ubiquitination required for targeting the M_1 _mAChRs toward down-regulation. We did observe increased constitutive down-regulation of the M_1 _mAChR with both mutant β-arrestin constructs. It is possible that the pattern of ubiquitination of these mutant β-arrestins altered the constitutive targeting of the M_1 _mAChR toward lysosomal down-regulation. This observation is one of several in this study that suggests that the pattern of ubiquitination on β-arrestin is directly implicated in the downstream targeting of mAChRs. Previously it has been suggested that the only role for β-arrestin ubiquitination is to promote the internalization of GPCRs.

We obtained surprisingly different results with the effects of the β-arrestin lysine mutants on the agonist-promoted down-regulation of the M_2 _mAChR subtype. Mutation of the two lysine residues in β-arrestin 2^K11R, K12R ^had no effect on either agonist-promoted internalization or down-regulation of M_2 _mAChRs. This result is similar to those seen with other Class B receptors such as the V2R and NK1R [22]. The results are different however from those seen with another Class B receptor, the AT1a receptor. When this receptor was co-expressed with β-arrestin 2^K11R, K12R^, agonist stimulation resulted in transient association of the receptor with β-arrestin 2, thus converting the receptor to a Class A type.

In contrast, the β-arrestin 2^K18R, K107R, K108R, K207R, K296R ^mutant significantly reduced M_2 _mAChR degradation with no effect on receptor internalization. These results are similar to those seen with the Class B V2R, where β-arrestin 2^K18R, K107R, K108R, K207R, K296R ^was shown to impair association of V2Rs with β-arrestin 2 on endosomes [22].

The fact that β-arrestin 2^K18R, K107R, K108R, K207R, K296R ^was able to internalize but not down-regulate the M_2 _mAChR suggests that one or more of these lysines has a role in down-regulation but not internalization for this subtype. These data suggest a direct role for the ubiquitination state of β-arrestin in the lysosomal targeting of the M_2 _mAChR.

Unlike effects observed with the M_2 _mAChR, β-arrestin 2^K18R, K107R, K108R, K207R, K296R ^was able to mediate down-regulation of M_1 _mAChRs. This observation clearly demonstrates that the role of these lysines in down-regulation is subtype specific and that a specific pattern of ubiquitination on β-arrestin can differentially target the two mAChR subtypes for down-regulation.

Co-localization of wild-type and β-arrestin 2^K11R, K12R ^with the M_2 _mAChR, observed after 30 min of agonist treatment is absent with β-arrestin 2^K18R, K107R, K108R, K207R, K296R ^which may indicate a disrupted or impaired interaction between this β-arrestin mutant and the M_2 _mAChR. β-arrestin 2^K18R, K107R, K108R, K207R, K296R ^was also shown to disrupt the co-localization of the M_2 _mAChR and the lysosomal marker LAMP-1 confirming our observation that β-arrestin 2^K18R, K107R, K108R, K207R, K296R ^does not mediate the agonist-promoted degradation of the M_2 _mAChR. Surprisingly, no co-localization of M_1 _mAChR was observed with any of the three β-arrestin constructs. Time points from 1 min to 12 hrs of carbachol treatment were performed to attempt to image co-localization between the M_1 _mAChR and the β-arrestins (data not shown). This observation seems to indicate that the role of β-arrestin in M_1 _mAChR down-regulation may involve a transient interaction between β-arrestin and the receptor which fates the receptor toward degradation.

Our observations of M_2 _mAChR trafficking are different from the results of work with the Class A β_2 _AR [17] which suggests that ubiquitination of β-arrestin is required for receptor internalization and that ubiquitination of the receptor itself is required for receptor degradation. Our data indicate that, for the M_1 _mAChR, the ubiquitination state of β-arrestin appears to have a role in the level of constitutive (agonist-independent) down-regulation and for the M_2 _mAChR the ubiquitination state of β-arrestin has a direct role in the agonist-promoted down-regulation of the receptor. Of the seven lysine residues we examined, at least one (or more) of the group 18, 107, 108, 207 and 296 are required for this M_2 _mAChR down-regulation. We also observed that M_2 _mAChR internalization was independent of β-arrestin ubiquitination at these same lysine residues that have been shown to interfere with the association of β-arrestin with other Class B receptors [22]. The possibility that other lysine residues than those we examined are ubiquitinated in order to internalize the receptor would preserve the role for β-arrestin in receptor internalization. The absence of down-regulation of M_2 _mAChRs with β-arrestin 2^K18R, K107R, K108R, K207R, K296R ^however, clearly indicates a direct role for the ubiquitination of β-arrestin in receptor down-regulation.

## Conclusion

We conclude that agonist-promoted down-regulation of both M_1 _and M_2 _mAChRs is β-arrestin-dependent. We further conclude that this down-regulation is specifically modulated by the ubiquitination state of β-arrestin and that this β-arrestin ubiquitination differentially targets the receptor subtypes for degradation in lysosomes. Significantly, where it has been suggested that Class A GPCR down-regulation proceeds primarily via receptor ubiquitination, these data indicate that the ubiquitination state of β-arrestin 2 has an essential role in the differential targeting the M_1 _and M_2 _mAChR toward down-regulation. Future studies will examine the role of receptor ubiquitination in M_1 _and M_2 _mAChR down-regulation. Specifically, we will examine whether expression of wild type *vs *the β-arrestin 2^K18R, K107R, K108R, K207R, K296R ^mutant differentially affects M_1 _and M_2 _mAChR ubiquitination and subsequent down-regulation.

## Methods

### Reagents

[^3^H]-*N*-methylscopolamine -(81–84 Ci/mmol) and [^3^H]-L-quinuclidinyl benzilate- (43 Ci/mmol) were purchased from Amersham Corporation (Buckinghamshire, England). LipofectAMINE 2000 and Protein A agarose were purchased from Invitrogen (Carlsbad, CA). Halt protease inhibitor and BCA protein assay kit were purchased from Pierce (Rockford, Il). Atropine, N-ethyl maleimide, carbamyl choline chloride, -anti-FLAG polyclonal antibody (F7425); polyclonal anti-LAMP1 antibody (L1418) and all other chemicals were purchased from Sigma Aldrich (St. Louis, MO). Anti-FLAG monoclonal antibody (200–472) was purchased from Stratagene (La Jolla, CA). Monoclonal anti-HA antibody (MMS101R) was purchased from Covance (Berkeley, CA). Polyclonal anti-HA antibody (71–5500) was purchased from Zymed (Carlsbad, CA). Monoclonal anti-Ub antibody P4D1 (sc 8017) was purchased from Santa Cruz Biotech (Santa Cruz, CA). Alexa 594 goat anti-mouse, Alexa 488 goat anti-Rabbit and Texas red goat anti-mouse (115-075-003) were purchased from Molecular Probes (Eugene, OR). FITC goat anti-rabbit (AP132F) was purchased from Chemicon (Temecula, CA). -HRP-conjugated secondary antibodies were purchased from Bio-Rad Laboratories (Hercules, CA). Prolong Gold Antifade (P36930) was purchased from Jackson Immuno Research (West Grove, PA.). pIRESeGFP were purchased from Clontech (Palo Alto CA).

### Expression constructs and cell lines

Expression constructs and cell lines were generously provided by the following: MEF wild-type cells, β-arrestin 1 and 2 double knockout cells, FLAG-tagged β-arrestin 1 and 2, YFP-β-arrestin-2-Ub, β-arrestin 2^K11R, K12R^, and β-arrestin 2^K18R, K107R, K108R, K207R, K296R ^(pcDNA3.1 FLAG β-arrestin 1 or 2, pEYFP β-Arr-Ub, pcDNA3.1 FLAG β-arrestin 2^K11R, K12R^, and pcDNA3.1 FLAG β-arrestin 2^K18R, K107R, K108R, K207R, K296R^) by Dr. Robert Lefkowitz (Duke University Medical Center, Durham, NC); HA tagged human M_2 _mAChR (pRK HA M_2 _mAChR) by Dr. Audrey Claing (University of Montreal, Montreal Canada); eGFP tagged M_1 _mAChR (pCEP4eGFP M_1 _mAChR by Dr. Brigitte Ilien (University of Strasbourg, Strasbourg, France).

### Cell culture and transient transfection

Mouse embryonic fibroblast (MEF) cells (wild-type and knockouts) were maintained in Dulbecco's Modified Eagle's Medium (DMEM). Media was supplemented with 10% fetal bovine serum (FBS), 100 IU/mL penicillin, and 100 μg/mL streptomycin. All cells were maintained at 37°C with 5% CO_2_. At 24 hr prior to transfection, cells were plated at 7.5 × 10^4 ^cells/well (12-well plate), 1.5 × 10^5^cells/well (6-well plate) and 2 × 10^6 ^cells/dish (100 mm dish). Cells were transfected using LipofectAMINE 2000 according to the manufacturer's protocol with 1.6, 5 or 25 μg total DNA per well/plate on a 12-well plate, 6-well plate and 100 mm dish, respectively. Transfection efficiencies of 40 – 60% were routinely obtained [determined by including 10% of total DNA as eGFP construct (pIRESeGFP) and visualizing transfected cells using an Olympus 1X71 fluorescent microscope].

### Crude membrane preparation

Two wells of a 6-well plate were rinsed twice with ice cold PBS and cells were scraped in 50 mM sodium phosphate pH 7.0, pooled and homogenized with 20 strokes through a Dounce homogenizer. Homogenate was spun at 10,000 rpm for 20 min at 4°C in a Sorvall Mach 1.6R fixed angle rotor. Pellet was resuspended in 50 mM sodium phosphate pH 7.0 and spun again. Pellet was resuspended in 0.55 mL 50 mM sodium phosphate pH 7.0 and used for protein assay and radioligand binding.

### Immunoprecipitation

At 24 hr post transfection, cells were lysed by sonication in HEPES lysis buffer (50 mM HEPES, pH 7.5, 1% Triton X-100, 10% glycerol, 10 mM NaCl, 1 mM EDTA, 1 mM EGTA, 50 mM NaF, 10 mM N-ethyl maleimide, 1 μg/mL each leupeptin, aprotinin, and pepstatin A). Next, 500 μg (BCA) of lysate was precleared using Protein-A agarose beads for 4 hr at 4°C followed by incubation with 5 μg of monoclonal anti-FLAG antibody at 4°C overnight. The immunocomplex was incubated for 4 hr with 100 μL Protein-A beads (50% slurry) at 4°C with rotation. Beads were washed 3 × 500 μL in the same buffer and resuspended in 30 μl of Laemmli buffer and boiled for 5–10 min. The immunocomplex was resolved on a 10% sodium dodecyl sulfate polyacrylamide gel electrophoresis (SDS-PAGE) gradient gel.

### Immunoblotting

Lysates were prepared as described above and 10 – 20 μg total protein was run on a 10% SDS-PAGE gel. Samples from immunoprecipitation or for direct immnuoblotting were transferred to nitrocellulose membranes, blocked with 5% powdered nonfat milk in Tris Buffered Saline, 0.1% Tween 20 (TBST) and probed with antibody as indicated. Immunoreactive bands were visualized with a Fuji imaging system using enhanced chemiluminescence after adding HRP conjugated secondary antibody.

### Radioligand binding

#### Internalization

Internalization was determined by measuring the binding of – the membrane-impermeable muscarinic antagonist [^3^H]-*N*-methylscopolamine (NMS) to intact cells. Briefly, 24–42 hr after transfection, MEF cells were treated or not treated with 1 mM carbachol for 30 min or 1 h at 37°C. Cultures were washed 3 × 1 mL with serum free media and incubated with 100 nM – [^3^H]-NMS in 1 mL PBS for 30 min at 37°C or 4 hr at 4°C. Nonspecific binding was determined as the bound radioactivity in the presence of 1 μM atropine. Labeled cells were washed 3 × 1 mL with ice-cold PBS, solubilized in 0.5 mL 1% Triton-X-100, and combined with 3.5 mL scintillation fluid followed by measurement of radioactivity. Cpm were converted to receptors per well which were then corrected to receptors per cell by dividing by cells/well. Cells were counted using a standard haemocytometer. Receptor internalization is defined as percent of surface M_2 _mAChR not accessible to [^3^H]-NMS at each time relative to untreated or control cells.

#### Down-regulation

Down-regulation was determined by measuring the binding of the membrane permeable muscarinic antagonist [^3^H]-L-quinuclidinyl benzilate (QNB). Crude membranes (100 μL) were incubated with 30 nM [^3^H]-QNB in a total volume of 1 mL 50 mM sodium phosphate pH 7.0 for 2 hr at 25°C. Nonspecific binding was determined as the bound radioactivity in the presence of 1 μM atropine. Membranes were harvested on glass fiber filters using a Brandel cell harvester (Gaithersburg, MD) and washed 3 × 2 mL with ice cold 50 mM sodium phosphate pH 7.0. Filter discs were combined with 3.5 mL scintillation fluid followed by measurement of radioactivity.

Cpm per tube was converted to fmoles receptor, which was then corrected to mg of total protein per tube. Percent down-regulation was determined as percent of sites remaining compared to untreated control membranes.

### Indirect immunofluorescence

#### LAMP-1:M2 mAChR co-localization

Cells were fixed in 4% formaldehyde in PBS for 5 minutes, and rinsed with 10% fetal bovine serum and 0.02% azide in PBS (PBS/serum). Fixed cells were incubated with primary antibodies (1:1000 anti-LAMP1 and 1:500 anti-HA MAb) diluted in PBS/serum containing 0.2% saponin for 45 minutes, and then washed with PBS/serum (3 × 5 min.). The cells were then incubated with fluorescently labeled secondary antibodies (1:800 Alexa 488 goat anti-rabbit and Alexa 594 goat anti-mouse) in PBS-serum and 0.2% saponin for 45 minutes, washed with PBS/serum (3 × 5 min.) and once with PBS, and mounted on glass slides in prolong gold antifade.

#### FLAG-β-Arrestin 2:HA-M2 mAChR or eGFP-M1 mAChR co-localization

Cells were fixed in 4% formaldehyde in PBS for 5 minutes. Fixed cells were permeabilized for 5 minutes with 0.1% triton in PBS and washed 3 × with PBS and blocked in rabbit pre-immune sera (1:200 in PBS). Fixed cells were incubated with primary antibodies (1:500 anti HA PAb or 1:800 anti FLAG MAb) rinsed 3 × with PBS ad incubated with fluorescent secondary antibodies (1:1000 FITC goat anti-rabbit or 1:1000 Texas red goat anti-mouse). Coverslips were mounted on slides in prolong gold antifade. Images were acquired using an Olympus 1X71 inverted fluoresecent microscope equipped with a 60x oil immersion objective. Images were obtained with Olympus Image Manager (Center Valley, PA).

## Abbreviations

AT1aR: angiotensin 1a receptor; β_2 _AR: β_2 _adrenergic receptor; CHO: Chinese hamster ovary cells; GPCR: G protein-coupled receptor; MAb: monoclonal antibody; mAChR: muscarinic acetylcholine receptor; MEF: mouse embryonic fibroblast; NK1R: neurokinin 1 receptor; NMS: *N*-methylscopolamine; PAb: polyclonal antibody; QNB: quinuclidinyl benzilate; V2R: vasopressin 2 receptor.

## Competing interest Statement

The authors declare that they have no competing interests.

## Authors' contributions

VAM performed down-regulation, internalization, immunoblotting, participated in the design of the study and drafted the manuscript. KJTperformed down-regulation and immunocytochemistry. KMH performed immunocytochemistr. NAM was involved in critical revision of the manuscript. DAJ designed the study and participated in drafting and critical evaluation of the manuscript. All authors read and approved the final manuscript.
